# The beginning of the end: A qualitative study of falls among HIV+ individuals

**DOI:** 10.1371/journal.pone.0207006

**Published:** 2018-11-08

**Authors:** Julie A. Womack, Gina Novick, Terri Fried

**Affiliations:** 1 VA Connecticut Healthcare System, West Haven, CT, United States of America; 2 Yale School of Nursing, West Haven, CT, United States of America; 3 Department of Internal Medicine, Division of Geriatrics, Yale School of Medicine, New Haven, CT, United States of America; University of South Florida, UNITED STATES

## Abstract

Falls are an important concern for individuals living with HIV (HIV+). The purpose of this study was to understand perceptions of HIV+ individuals who had fallen regarding what caused their falls, prevention strategies that they used, and the impact of falls on their lives. Qualitative Description was the approach best suited to our study. We conducted in-depth interviews with 21 HIV+ individuals aged 47 to 71 years who had fallen within the past two years and who received care in a primary care/HIV clinic. Participants identified causes of falls as intrinsic (HIV, opportunistic infections, antiretroviral therapy, substance use, polypharmacy) or extrinsic (icy sidewalks, wet floors). Among those who felt that their falls could be prevented, prevention strategies included physical therapy and avoiding extrinsic fall risk factors. Some participants, however, felt that their falls could not be prevented. While some participants responded adaptively to falls, for many, the experience of falling was connected with deep feelings of loss and suffering. For these individuals, falls were understood to be “the beginning of the end” and a source of social isolation, changing family roles, diminished sense of self, and stigma.

## Introduction

Falls are an important concern among HIV infected (HIV+) individuals in part because of their prevalence and because of the negative outcomes associated with them. One study found that the prevalence of falls was 30% among HIV+ individuals 45–65 years of age [1], similar to the 25–45% prevalence identified in community dwelling older adults 65+ years of age [2]. Preliminary research from our group suggests that among HIV+ individuals, falls are associated with an increased risk of injury and mortality, much as they are in older adults in the general population.

This work makes clear the need for fall prevention programs. Whether these interventions should be the same as those tailored to the needs and perspectives of older adults is not known. To develop strategies for fall prevention among HIV+ individuals, we first need to understand how HIV+ individuals’ perceptions regarding the causes of their falls, what they do for prevention, their perspectives on their providers’ response to their falls, and the impact of falls on their lives and sense of self.

There has been extensive research on falls among older adults (aged 65+ years) in the general population. Important fall risk factors include cognitive impairment, disability of the lower extremities, abnormalities of balance and gait, polypharmacy, sedative use [3], dehydration [4], digoxin, antiarrhythmics and diuretic use [5]. In qualitative studies, older adults reported being afraid of falling as falls are associated with adverse outcomes such as injury, hospitalization, and death. Many also viewed fall prevention efforts as ineffective or as not being applicable to their specific situation [6].

Fall research in the HIV+ population identified multimorbidity, polypharmacy, and functional impairment as important fall risk factors [1]. In addition to the total number of medications prescribed (polypharmacy), specific medications were associated with falls, particularly those that were active in the central nervous system (CNS) such as opioids, muscle relaxants, and benzodiazepines [7, 8]. Frailty and peripheral neuropathy have also been associated with falls in this population as have symptoms of dizziness or imbalance [9–11]. HIV-specific risk factors such as CD4 count and HIV-1 RNA may be associated with falls, but results are not consistent across cohorts [1, 12]. This area requires further research.

There has been minimal exploration of HIV+ individuals’ perspectives on their falls. Many of the HIV+ individuals who fall are relatively young (typically less than 65 years), thus the results of prior studies examining older adults’ perspectives on falls may not apply [2, 3]. For example, because they are younger, HIV+ individuals may not consider themselves to be at risk for falls or for adverse fall outcomes such as fractures or traumatic brain injury. Consequently, fear of falling might not be as important an aspect of their experience of falls as it is among older adults [13] The medical, social, and psychological implications of living with HIV may also influence the perceived experience of falls. For example, relative to older adults, fall-related stigma may be more important among HIV+ individuals, an already stigmatized group. Finally, there may be differences in how providers respond to falls. The association between falls and negative outcomes including serious injury, hospitalization, and death in older adults is well-established [14]. An awareness of these outcomes drives provider concern about falls in their older patients. These outcomes are just beginning to be identified among younger, HIV+ individuals who fall. Providers may not be aware of these negative associations and thus may not be concerned about the adverse outcomes of falls in this population.

We therefore sought to develop an understanding of the experience of falls among HIV+ individuals. We were interested in exploring their perspectives on the causes of falls, provider response to falls, what can be done to prevent falls, and the impact that falls have on their lives.

## Materials and methods

### Design

Qualitative Description [15, 16] was the approach best suited for our study as it allowed for a rich description of participant experience depicted in easily understood language [17]. The outcome of Qualitative Description should be a clear description of the participants’ experiences that provide clinicians with an understanding of how to improve the healthcare of the population of interest [15, 17]. We wanted to remain close to participants’ words, capturing and describing events and meanings as participants understood them. We anticipate that our findings will be used to develop fall prevention programs that target the needs and concerns of this population. No specific analytic techniques are prescribed by Qualitative Description. Sampling, data collection, analysis, and presentation techniques were selected to fit the research questions and the needs of the study [16].

The authors brought a variety of clinical experience and qualitative research expertise to the project. The first author is an expert HIV clinician and researcher who has been trained in qualitative research methodology. She maintains a clinical practice in the clinic from which participants were recruited. The second and third authors are expert qualitative researchers who have published many qualitative studies. Their clinical areas of expertise include women’s health (GN) and geriatrics (TF).

### Participants and setting

We recruited HIV+ men and women from an HIV primary care clinic in an urban, northeastern setting. This clinic reaches a diverse population in terms of age, ethnicity, living situation, and experience of HIV infection. Because there have been no in-depth qualitative studies published on the experience and impact of falls on the lives of HIV+ individuals, we wanted to include perspectives from as wide a variety of participants as possible. To gain maximum variation in our sample, we began with consecutive sampling and screened every patient who presented to clinic. Seeing that this approach was successful in achieving a diverse sample, we continued with it for the duration of study recruitment (10 months).

Our study was open to all patients who presented to clinic and who had fallen within the past two years. Patients with falls more distant than two years were not included because of concerns regarding recall bias [18]. Exclusion criteria included a diagnosis of dementia or being otherwise incapable of consenting to participate in the study.

Participants were recruited in one of two ways: through the routine clinic screening process, or in response to fliers posted around the clinic. As part of the routine clinic screening process, the clinic staff member who conducted the screening inquired whether patients had fallen in the past two years. If the patient responded affirmatively, the staff member described the study to the patient and asked if they would be willing to be contacted by study personnel. If patients agreed, they were asked to provide contact information and to sign a statement agreeing to be contacted by study personnel. Study personnel then reached out to patients by phone to provide them with additional information about the study. If the patient was interested in participating, an appointment for the interview was scheduled, and verbal consent was requested to review their electronic health record. To enhance sample size and variation, we expanded our recruitment efforts by posting fliers in the clinic with information about the study. These fliers included contact information for study personnel so that patients could contact them directly without involving clinic staff. All participants were provided with information on the reasons for the study and how the information gathered would help expand what is known about falls among HIV+ individuals.

We continued recruitment until thematic saturation was achieved [19, 20]. Thematic saturation was identified when we found that additional interviews provided only additional instances of themes already identified but did not help to expand or elaborate on existing themes [21].

Data were collected from in-depth, semi-structured interviews and from review of participants’ electronic health records. The interviews provided the data central to our study aims. Chart review provided demographic and background clinical information. Duration of HIV infection was provided by self-report and confirmed by chart review.

### Procedures

Once participants agreed to participate in the study, they were offered the option of a face-to-face or a telephone interview [22]. Those who wished to have face-to-face interviews decided where they wanted to be interviewed: in their home, in their office, or in a private room at the medical center.

The first author conducted in-depth, semi-structured interviews. We first asked patients to focus on the index fall: the worst fall that they had experienced in the last two years and since being diagnosed with HIV. Our discussion began with this fall, but participants could talk about any other falls that they felt were significant or important. The prompts for the interview can be found in Table 1.

**Table 1:** Prompts for semi-structured interviews.

Components	Details
Introduction	I am going to be asking you some questions about the worst fall that you have experienced in the last two years and since you were diagnosed with HIV. We’ll be talking about what you thought about the fall, what you think caused your fall, how you think it could have been prevented, and some things you can do to prevent falls—things that you’ve done, things that others have told you about. I’m also interested in hearing about what helps and what gets in the way of doing these things. I’m really interested in your views about your fall–so if there are thoughts or experiences that you would like to share with me, even if I haven’t asked about them, please feel free to do so. You may refuse to answer any of the questions that I ask, and you may end the interview at any time for any reason. I want to emphasize that this is not a clinic visit. You should not feel that you need to keep your answers short. I want to hear what you have to say, so we have as much time as we both feel we need for you to talk about your experience. Do you have any questions before we begin? As we discussed, I’ll be recording the interview.
Interview prompts	
Type of interview	When we first spoke, I offered you the option of a face-to-face interview or a telephone interview. Can you tell me why you chose to do the interview either face-to-face or by telephone?
Description of the index fall	Tell me about the fall that you had on __(date)_______________. Prompts: 1. When did this fall occur? 2. Where did this fall take place? 3. Please describe what happened: a. Tell me about what was going on right before you fall: i. What were you doing right before the fall? ii. What do you think caused the fall? b. Describe what happened right after the fall i. What happened when you tried to stand up or move? Was this difficult? ii. Who was around when you fell? Can you describe what, if anything, they did to help you? iii. If someone wasn’t around, what did you do? 1. How long before someone came to help you? iv. How much time passed before you could get up or before someone came to help you? c. Once you got up, what happened next?
Severity of the fall	What was it about this fall that made you go in to see your provider or go to the emergency room? Was this the worst fall that you’ve experienced? Why or why not?
How has the fall impacted your life	1. How have you modified what you do every day because of this fall? 2. How has it changed how you think about routine daily activities: a. Getting into the bathtub? b. Getting out of bed? c. Walking up the stairs? d. Walking alone? e. Navigating the bus?
At risk for future falls?	Can you talk about why you do or do not think that you are at risk for having another fall. Prompts: 1. Are there things about your life that you think might cause you have another fall? 2. Can you describe ways in which your health might put you at risk for another fall? a. Medications? b. Were you sick at the time? c. Do you need to get up frequently at night? d. HIV?
If you have had other falls	1. Have you had other falls before or after this one? Can you tell me about them?
Fall prevention	1. What do you do to prevent yourself from having another fall? 2. Have you heard about other things that you can do that might help prevent a fall? Specifically things that you haven’t tried? a. Have friends suggested anything? b. Health care providers? 3. What do you think would be most helpful? 4. Have you tried any of these things? What happened? a. Other things to ask about if they don’t mention them: i. Exercises to keep you from getting dizzy when you stand up or change position quickly. ii. Exercises to help you improve your balance. iii. Exercises to strengthen your arms and legs. iv. Elevate the HOB. v. Discontinue medications that help you sleep or that calm you down. vi. Decrease the number of medications that you take. vii. Make modifications to your bathroom to make getting on and off the toilet or into or out of the tub easier. viii. Work with you to make your gait steadier. 5. What gets in the way of doing what you need to do to prevent falls? 6. What helps you do what you need to do to prevent falls?
Importance of falls	1. How important of a problem is falls and falls prevention for you?
Additional questions	1. We’ve had an extensive discussion, but is there anything else that I should be asking? Anything that we haven’t talked about that you would like to mention? 2. Finally, can you tell me how you found the interview experience, particularly, how was it doing the interview (face-to-face OR by telephone)? What was good about it? What was bad?

The interviews were digitally recorded and professionally transcribed verbatim. Transcripts were then uploaded to ATLAS.ti (version 7.5.18). The interviewer took field notes during and immediately after the interviews to record key thoughts and impressions about the interviewee and the interview process. Interviews were conducted between February and November 2013. We also reviewed each participant’s electronic health record to capture demographic and additional healthcare data including information on comorbidities and medications.

### Analysis

Analysis began during data collection, allowing the data gathered to inform study design. This approach is consistent with emergent design where the study design evolves and can be modified as data are collected [23, 24]. Emergent design can be used by many different qualitative approaches (e.g. grounded theory, phenomenology, qualitative description.

The first and second authors read all of the interviews and developed a start list of codes [23] derived from the study’s specific aims, relevant literature, and interview probes (Table 1) [25]. As transcripts were completed, the first author coded them using these as well as additional inductive codes (codes that emerged from the interviews). After the first five interviews were coded, all codes, along with corresponding interview data, were reviewed by the first and second authors. The authors developed a coding structure that integrated the preliminary and inductive codes. The first author then recoded the interviews using these revised codes. This process continued until all interviews were completed and coded. At that time, five coded interviews (20% of all interviews) were randomly selected to be coded by the second author. The first and second author then compared the coding of the five documents to explore similarities and differences in code structure and content. Consensus was used to further develop and refine the final codes. All interviews were then recoded by the first author using the finalized codes.

Through working with the individual interviews and developing a coding schema, the first author determined initial conceptual groupings of the data and identified preliminary key ideas or themes. Codes that were relevant to these preliminary themes were identified, and the data associated with these codes were examined for patterns. Data for the remaining codes were then examined to identify additional themes as well as negative cases that challenged the existing themes. Content was compared across codes to seek common ideas and to identify and refine the themes. Themes and supporting data were then reviewed by the second author who made suggestions for alternative interpretations and suggested additional themes. Final themes were identified by consensus.

### Rigor

In qualitative research, rigor is defined as trustworthiness [26], consisting of credibility, transferability, dependability, and confirmability. Strategies to support credibility include triangulation of data from different sources, peer debriefing, and negative case analysis. In addition to interview data, we also included data from each participant’s medical record. The first and second authors analyzed the data separately and came to a consensus on their interpretations. Analysis of negative cases expanded the themes identified in our study. Transferability involves adequate description of the participants, their data, and the context. Readers can then evaluate how well the data and our interpretation can be applied to different individuals in different contexts. We provided information about the participants (Table 2) and referred to this information when using quotations in the manuscript. Dependability is associated with credibility and maintenance of a careful audit trail. The study and its findings are auditable: we have preserved research memos, data display charts, coding instructions, and documentation of our process for developing themes [18]. Cross-checking coding and themes added to the reliability of the findings [27, 28]. Confirmability is achieved through the use of more than one data source and through maintenance of a reliable audit trail, both of which are described above. We also adhered to the Consolidated Criteria for Reporting Qualitative Research (COREQ) (S1 Table).

**Table 2:** Characteristics of study participants.

ID	Age (years)	Sex	Race	Ethnicity	Employment	Year of HIV diagnosis	AIDS diagnosis	Gait impairment /requires use of assistive device(s)	Number of chronic comorbi-dities	Number of medi- cations	Falls are ongoing or in the past	Fall sequelae—physical
1	53	M	Black	Non-Hispanic	Unemployed	2004	Yes	Yes	10	18	Ongoing	Bumps/ bruises
2	49	F	White	Non-Hispanic	Unemployed	1988	No	No	12	8	Ongoing	Fractures, hospitaliz-ation
3	48	F	White	Hispanic	Employed	1989	Yes	No	5	3	Ongoing	Multiple fractures
4	49	F	Black	Non-Hispanic	Unemployed	Unsure	No	Yes	11	24	Ongoing	Bumps/ bruises
5	56	M	White	Non-Hispanic	Unemployed	1986	Yes	Yes	16	22	Ongoing	Hip fracture
6	56	F	Black	Non-Hispanic	Unemployed	2011	Yes	Yes	4	10	Past	Hospitaliz-ation
7	60	M	White	Non-Hispanic	Employed	1985	Yes	Yes	14	16	Ongoing	Bumps/ bruises
8	52	F	White	Hispanic	Unemployed	1992	No	Yes	9	13	Ongoing	Bumps/ bruises
9	57	F	Black	Non-Hispanic	Unemployed	2010	No	Yes	14	9	Ongoing	Joint swelling, bruises
10	54	M	Black	Non-Hispanic	Unemployed	Unsure	No	Yes	9	9	Ongoing	Hospitaliz-ation
11	71	F	Black	Non-Hispanic	Unemployed	1989	Yes	Yes	12	14	Ongoing	Bumps/ bruises
12	47	F	Black	Non-Hispanic	Unemployed	2012	Yes	Yes	10	14	Ongoing	Bumps/ bruises
13	62	F	Black	Non-Hispanic	Retired	1993	Yes	Yes	13	12	Ongoing	Bumps/ bruises
14	59	M	Black	Non-Hispanic	Unemployed	1991	No	No	8	17	Ongoing	Serious cuts/ laceration, head injury
15	55	F	Black	Non-Hispanic	Unemployed	1984	No	Yes	14	16	Ongoing	Fracture, hospitaliz-ation, lacerations
16	51	F	Black	Non-Hispanic	Unemployed	2008	Yes	Yes	11	18	Ongoing	Bumps/ bruises
17	62	F	White	Non-Hispanic	Employed	1986	No	No	6	11	Ongoing	Bumps/ bruises
18	50	M	Black	Non-Hispanic	Unemployed	1986	Yes	Yes	10	17	Ongoing	Fractures, lacerations, dislocation
19	53	M	White	Non-Hispanic	Unemployed	1990	Yes	No	26	29	Ongoing	Bumps/ bruises
20	52	M	Black	Non-Hispanic	Unemployed	2007	No	No	4	5	Ongoing	Bumps/ bruises
21	58	M	Black	Non-Hispanic	Unemployed	1980	No	No	7	8	Past	Bumps/ bruises

### Ethics

Internal Review Board (IRB) approval for this study was first obtained from Yale University Institutional Review Board in 2012 and has been renewed annually since then. Verbal or written informed consent, depending on whether the interview was conducted by telephone or face-to-face, was obtained from each participant.

## Results

We interviewed 21 participants: 16 were recruited through clinic screening, and five from direct response to posted fliers. Four individuals were approached by clinic staff but refused to participate; two agreed to be contacted but never responded to outreach. Among the four individuals who refused to participate, two cited time constraints and two said that they were not interested in the study. Each interview lasted between 33 and 136 minutes. The average duration of the interviews was 46 minutes. Eleven participants elected to have telephone interviews and ten elected face-to-face interviews. Of the face-to-face interviews, four occurred in participants’ homes, one occurred in the participant’s office, and five occurred in private offices in the medical center. No non-participants were present for the interviews. We conducted one interview per participant.

Participants’ mean age was 55 years (range 47 to 70) (Table 3). Nine of the participants were men, 14 were Black, seven were White and two were of Hispanic ethnicity. Most were insured by Medicare or Medicaid. Only two participants had private insurance. Participants had lived with HIV for an average of 19 years (range 1–33 years). Eleven had an AIDS diagnosis. Excluding a diagnosis of HIV which was common to all, multimorbidity (at least 2 chronic conditions) was identified in all participants. All participants reported having fallen multiple times. Two participants had stopped falling by the time they were interviewed but reported that their last fall occurred between six months and two years prior to the interview. All other participants reported that their falls were ongoing: their most recent having occurred within the six months prior to the interview. Fall-related outcomes ranged from bumps and bruises to hip fractures, multiple fractures, and hospitalization. Detailed information about the participants is provided in Table 2.

**Table 3:** Demographic and clinical characteristics of participants (N = 21).

**Demographic Characteristics**	N (%)*
Age (mean, standard deviation)	55 (6)
Women	12 (57)
Men	9 (43)
Black	14 (67)
**White**	7 (33)
Hispanic	2 (10)
**Non-Hispanic**	19 (90)
Employed	3 (14)
**Retired**	1 (5)
**Unemployed**	17 (81)
Lives with family	9 (43)
**Lives alone**	9 (43)
**Lives in group home**	3 (14)
Clinical Characteristics	
Year of HIV diagnosis (range)	1980–2012
AIDS diagnosis	11 (52)
Gait impairment/use of assistive device(s)	14 (67)
Number of chronic comorbid conditions (mean, standard deviation)	11±5
Number of medications (mean, standard deviation)	14±6

* except where noted otherwise

### Themes and sub-themes

**Fig 1:**
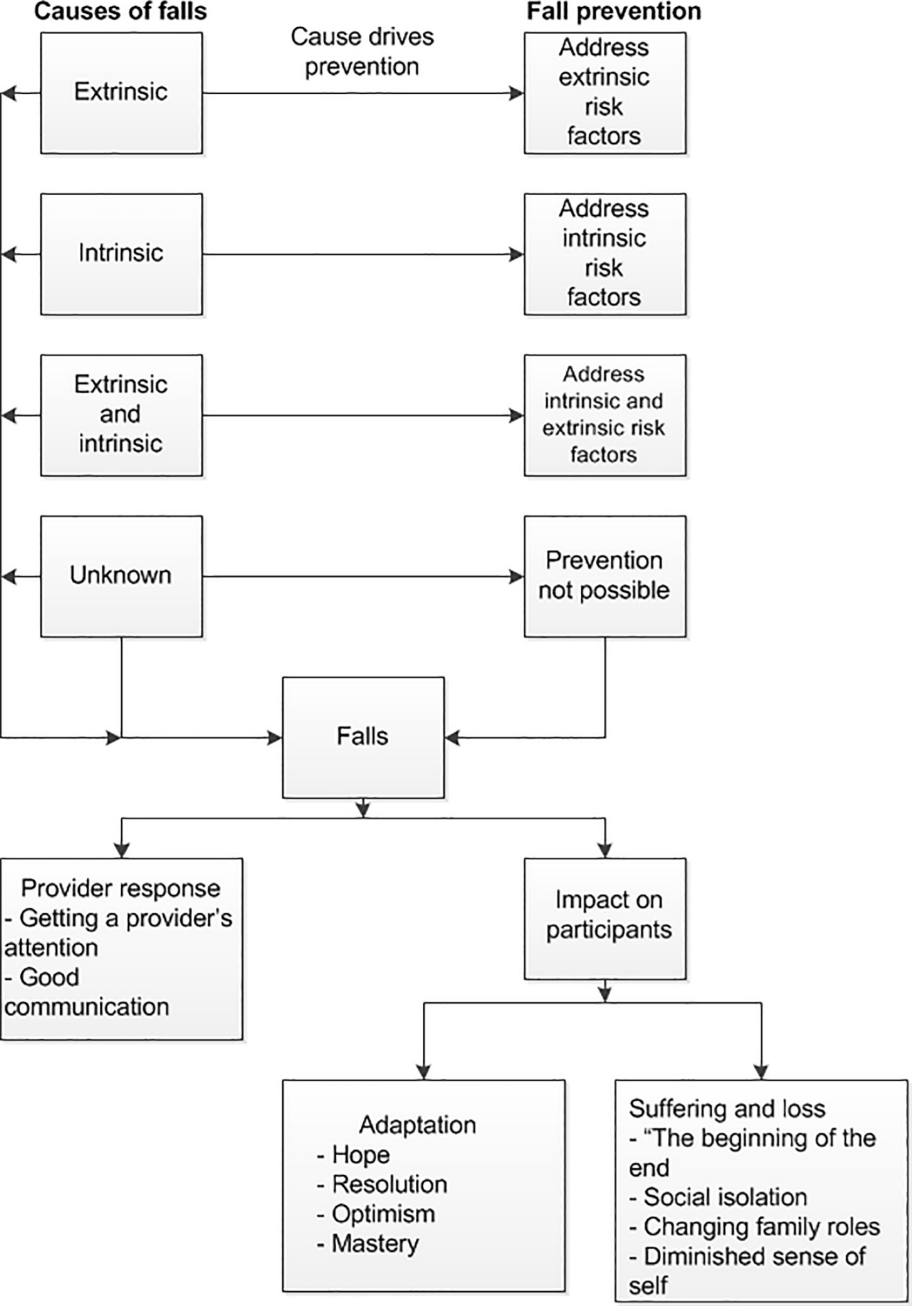
Relationship among areas of interest and study themes.

The topics that we addressed were: causes of falls, patients’ perceptions of provider response to falls, fall prevention, and impact of falls on participants’ lives. These key areas are noted in bolded text. Important themes within the areas of interest are noted in bolded text at the beginning of a paragraph, and subthemes are noted in italics. These areas of interest and the themes associated with them are summarized in Fig 1.

### Causes of falls

Participants identified a number of causes for their falls. We classified these causes as extrinsic (provoked by external factors only, such as slipping on ice or tripping on a rug), intrinsic (provoked by factors internal to the participant such as HIV, alcohol use, polypharmacy, or multimorbidity), a combination of extrinsic and intrinsic, or unknown.

#### Extrinsic causes

A few participants attributed their falls to extrinsic factors: two participants slipped while walking on icy streets, one was hiking and fractured her ankle when she tripped, one slipped on a wet tile floor, one fell while transferring from his wheelchair to his bed, and one was rushing up her apartment stairs while carrying too many groceries.

#### Intrinsic causes

Others described their falls as being driven by intrinsic causes including spinal stenosis, dehydration, arthritis, peripheral neuropathy, pain, hepatic encephalopathy, the number of medications they were taking, and a history of stroke with resulting neurological deficits. When participants attributed the number of medications they were taking to their risk of falling, we asked whether or not there were specific medications that were particularly problematic. Most reported that they could not remember all of the medications that they took, but they felt that the number of medications, more than any specific medications, drove fall risk.

Although all participants were HIV+, perspectives on the role of HIV infection as a risk factor for falls varied. Among those who reported that there was an association, one woman (Participant (P) 3) had fallen many times after contracting HIV and told the story of her falls by reflecting on changes in her body wrought by HIV:

… I never broke anything (before I had HIV)… Then (after starting antiretroviral therapy [ART])…I was just beginning to … gain the weight around the middle… to get … lipodystrophy symptoms and … my legs were very, very thin, and I started to gain a lot of weight in my stomach… and I … fell … three times that summer… I was … out of balance. It felt like the middle of me was just propelling me forward … I was gaining the weight rather rapidly, I mean … within … three years, I literally gained… 65 pounds and my limbs got extremely thin…

Another participant related his falls to sequelae from neurological opportunistic infections, specifically toxoplasmosis and progressive multifocal leukoencephalopathy. Others speculated on the role that ART played. Some felt that the number of medications increased their fall risk, and, as ART increased the number of medications prescribed, they contributed to the risk for a fall. One participant (P7) speculated that his falls were a side effect of ART toxicity. He explained: “I was wondering if (my falls) were related to the HIV medications that I’ve been on for all these years…A lot of people did die [in the early years of the epidemic, and] those who chose to take medications died. So you get it in your head that the medications are toxic… side effects [like falls] are the common denominator”.

#### Combination of intrinsic and extrinsic causes

Most participants felt that their falls were caused by a combination of both intrinsic and extrinsic factors. One woman (P13) described walking across a busy street. Her severe arthritis made movement and balance difficult, and she had been prescribed a cane to help with her balance. The cane hit a bump in the road, causing her to lose her balance, “and if your body ain’t perfect to catch yourself, you’re going down… the thing is, if I could maneuver my hip better, then when I start to fall, I could brace myself… but I can’t because of the pain… So if I’m falling, there’s nothing I can do…”

Many participants remarked on the complexity of their falls and the multiplicity of factors that contributed to them. Typically, falls were not seen as a consequence of a single factor; rather they resulted from the interplay of a number of different factors. For example, taking several medications was an important fall risk factor, but the medications didn’t act alone. As one participant stated (P19): “… every time you take [a medication]… depending on what’s in your system and how much sleep you’ve had, the reactions [to the medication] can vary…”

#### Unknown causes

A minority of participants had no notion of what caused their falls. One participant with a very complex medical history (P5) stated: “… my situation is very complicated, and it would be really hard to trace any etiology or conclude anything [definitive about a cause] from my own incidents, even though falls keep happening.” Some could identify a very general cause,—impaired balance or equilibrium—but could not explain what caused them to lose their balance (P11): “I just lost my equilibrium… just went over… No warning, no pain…”

### Participants’ perceptions of provider response to falls

#### Getting a provider’s attention

Participants often struggled to get providers to pay attention to their falls, particularly when providers could not readily attribute the fall to an acute condition (e.g. stroke or neurologic opportunistic infection). Some participants felt that provider responses to falls were illustrative of a more general problem: that what was important to the participants was not prioritized by clinicians, at least in part because the causes of falls could not be quantified, and if they could not be quantified, they were not real. As one participant (P3) described:

If the clinical research and the clinical data don’t catch up with the symptoms [like falls], then you have to fight for your symptoms… you have to convince somebody [that your symptoms are real], and if it doesn’t show up in the blood work, then it’s pointless… it’s like when you take your car to the mechanic. If the stupid computer doesn’t say there’s something wrong with your car, then there’s nothing wrong with your car, even though your car is making every noise under the sun. And the mechanic says, “I’m sorry, but the computer says that you are fine.” And that’s how I feel about falls… if your T cells are fine, and your viral load is fine, … [you’re] ok.

#### Communication

Some participants reported that communication with providers around falls was often unhelpful. Information was not discussed in a way that helped the participant understand what caused the fall and how it might be prevented. As one participant (P4) described: “Well, they say things, but it’s not in layman’s terms. It’s all medical talk. So I can’t explain to you exactly what (caused my fall). You’d have to … talk to my doctor”.

In contrast, one participant had a supportive provider who worked with him to identify the causes of his falls. Once polypharmacy was identified, the participant decided to stop all of his medications (ART, sleep medications, and mental health medications). Within a week, he had stopped falling. At his next visit, he talked with his provider about which medications to restart and when to restart them. The provider understood how concerning falls were to him, and rather than chastising him for discontinuing his medications, she helped him strategize about restarting them in a way that would not increase his fall risk.

### Fall prevention

Participants identified prevention efforts targeting both extrinsic and intrinsic fall risk factors. Prevention efforts were undertaken only if the participant could identify a specific cause for their falls. For example, two participants felt that their falls were caused by their degenerating hip or knee joints and believed that joint replacement surgery was the only way to definitively resolve their falls.

#### Preventing falls with extrinsic causes

A number of different strategies were used to prevent falls caused by extrinsic risk factors including wearing proper shoes, cleaning up clutter in the home, and being careful when walking on ice. Participants used walkers, canes, walls, or bannisters to give them extra support so they wouldn’t fall. Making modifications to the bathroom, including adding grab bars and elevated toilets, were also helpful. Others reported just trying to be careful (P3): “I *think* when I walk… I think about *walking*… I look at the ground real careful… Like, really, like I *monitor* the ground the whole time. When I take the steps at the garage, I’m like ‘I better not fall… I better not fall.’”

#### Preventing falls with intrinsic causes

Others focused prevention efforts on intrinsic risk factors such as staying well-hydrated and taking medications as directed. Most participants felt that taking ART was an important component of fall prevention as it kept them healthy, and being healthy, kept them from falling. There were exceptions to this perspective. One participant felt that his falls were caused by medication interactions. He therefore stopped all of his medications, including his ART. He (P21) also stated:

Then I [got] concerned about my health, so I went back to my doctor and I asked to be put back on the [ART].

Another participant (P7) discussed his ambivalence towards ART. He acknowledged that he had done well on ART while others had not, and that side effects were common:

A lot of people, you know, that did die… those who chose to take medications [early on] did die. So you get in your head that the medications are toxic, and [the government] is putting them out there because there were a lot of gay people… You know, back in the day when the evangelicals were saying that [HIV] was God’s way of telling us that he hates gay people and so on. So there’s a lot of paranoia about the medications. I just went on and took my medications. I tried not to believe what everyone else was saying, because they were all dying and I wasn’t… [but] I really don’t know… every time I change my ART medications, I’ll try them, but they all have the same side effects, nausea, vomiting… so what do you do? What do you take? There’s lots of toxic medications, but is [ART] any worse than any other medication? … You know, I don’t know… I don’t think there have been adequate studies for the drugs we’re on [ART].

This nuanced understanding of the risks and benefits of ART was not verbalized by other participants.

A number of participants identified hip or knee pain related to arthritis as important causes of falls. Most of these participants had been told by their providers that joint replacement surgery of the affected joint was the best way to prevent falls. Some admitted that they were afraid of having surgery but were resigned to it as the only option to prevent falls and restore their quality of life.

A small subgroup of participants felt that physical therapy and exercise were key components of fall prevention. Participants noted that the quality of physical therapists varied widely. With a good therapist, physical therapy could be very helpful; “bad” therapists were virtually useless. One participant (P10) who had extensive experience with physical therapists described the difference between a good and bad therapist:

This new [physical therapist] I got? … she spent time with me… she was doing all of this stuff with the nervous system… [S]he tested these points… and could tell me where I was tight up and everything… And she sat there. She spent time with me. She did relaxation stuff… like guru stuff. You know, it just made me feel better. The other person was like, come in, all right do this, do that, sign this paper and that’s it… [Some] people care and [others] do not… Some people do their job … [while] others just think, ‘I have all these patients and I’m late.’ You get 15 minutes when [you’re] supposed to get an hour. So you get cheated.

Other prevention activities included not “messing around with drugs” or alcohol, suggesting an ideal of complete abstinence from these substances. Others felt that having to walk everywhere was associated with falls and having access to a medical cab for transportation to and from appointments decreased this risk.

#### Falls can be difficult to prevent when causes cannot be identified

Others felt that it was difficult to prevent falls because they were unpredictable, suggesting that the only way to prevent a fall was by intervening immediately before the fall occurred. Having warning signs before a fall (e.g. dizziness) gave participants time to prevent falls by sitting down or by leaning against a wall. However, as one participant (P20) reported, “I can’t really even say that [I can control my falls]. …Because … I don’t know when it’s going to happen… I might do good for like two or three days or so and then I may just decide to just get up and… I fall, I don’t know when that may happen. . . . It’s… scary”.

### Impact of falls on participants’ lives

Whenever participants fell, the immediate post-fall concern was whether or not they had injured themselves. As one participant (P5) stated, “… everything grinds to a halt until I figure out how bad the fall has been.” Beyond this acute assessment, there were two broad typologies of responses to falls. Falls that resulted from extrinsic causes, such as twisting an ankle on a rock or slipping on a patch of ice, were not perceived as threatening, even though these types of falls could result in serious injury. They were seen as “something that could happen to anyone,” suggesting that these falls did not set them apart from others who were not sick or who did not have HIV infection. For those who identified intrinsic causes, however, two main responses to falls were identified: some participants had adapted to their chronic illnesses and to falls as a symptom of these underlying conditions. Others were devastated and focused on the loss and suffering they experienced because of them. These responses did not differ by demographics, presence of an AIDS diagnosis, or year of HIV diagnosis.

#### Adaptation

Those who adapted to their falls and underlying chronic illnesses demonstrated several responses.

**Hope.** Some saw their falls and the conditions associated with them as transient or as something that could be corrected. They viewed their current fall-related disability as temporary. Looking forward to the fix generated hope and optimism. One participant eagerly anticipated knee replacement surgery. She attributed her falls with the pain caused by degenerative changes in her knee associated with osteoarthritis. She had been told by her providers that the best way to treat this pain was with joint replacement surgery. She also had a clear vision of her life after surgery and rehabilitation: she would find a place of her own (which she had never had) and believed she might be able to hold down a job for the first time in years. Rather than focusing on the pain and disability that she experienced in the present, the prospect of a fix helped her focus on a more positive future.

**Resolution of falls**. Two of the participants had stopped falling at the time of the interview, which likely explained their optimism. One participant started to fall shortly after she was diagnosed with HIV. Her falls resolved after starting effective antiretroviral therapy. Whereas she had expected to die and saw her falls as an outward sign of her despair, she discovered that she could live with HIV by taking her medications. The resolution of her falls was an important component of her return to health.

**Optimism**. Other participants felt that their health was worsening but nevertheless were able to maintain an optimistic outlook by comparing their current situation favorably with their past or with situations of those less fortunate. One participant (P16) noted: “I remember… when I first started getting arthritis in my knees [and] how frustrating it was for me to [fall and] be so limited all of a sudden… And so now I’m limited… [but] I have all of that wealth of experience to draw from. So, I don’t feel limited … because I look at the limitations differently. You learn to deal with stuff…” Similarly, another participant (P5) described having spent four years in the hospital with opportunistic infections and compared that experience to his current situation that included living with falls: “Compared to things that I’ve gone through, [the falls] are insignificant …” Another participant compared herself to friends or colleagues who were dealing with far more severe illnesses and limitations, saying “I know so many people who have been through so much, and they have a wonderful outlook on life”.

**Mastery.** Other participants demonstrated a sense of mastery. One participant’s identity was defined by his ability to manage his chronic illnesses. He was well read about his conditions and often had suggestions for his providers about new management options. He felt that his providers appreciated his input and respected his knowledge and his desire to be an active participant in his health care. When he started to fall, falls became another condition to be managed. He understood that he could not prevent all falls, so he learned how to “fall safely” (P19):

Thankfully, I had the sense to throw my weight down… You know, rather than to go with the momentum of… the fall. I just… threw my weight down towards the stairs… and I landed on my side [and not on my head].

#### Suffering and loss

The optimism and hope demonstrated by those who were able to adapt to their chronic illnesses and falls stood in marked contrast to the experience of suffering and loss described by others. For these latter participants, falls were associated with fear and indicated deteriorating health, changed social and family roles and relationships. Falls threatened their identity and their sense of self. In this section, we describe five broad typologies of responses to falls characterized by loss and suffering: “the beginning of the end,” social isolation, changing family roles, diminished sense of self, and stigma.

**“The beginning of the end.”** Falls were often viewed as a portent of worsening illness and a marker of “the beginning of the end.” For these participants, fear was an important component of their response to falls. Falls were a marker of deterioration, decline, disability, and the loss of one’s previous life of independence and agency. As one participant (P3) described,

I'm always worried about [falls]… Because you know what? It takes away your life… it reminds me about …what it would be like to be disabled, what would it be like [to] get to the point that… you can’t walk very well. It’s not about age, it’s… illness. [Falls] remind me of illness and …[not] being able to take care of yourself and becom[ing] dependent…

Although some participants did not associate AIDS with falls, they still saw their falls as a marker of chronic illness, declining health, and an increased risk of death (P15): “I’m getting older, and as I think about getting older, I equate that with my mortality. So [falling includes] a degree of fear… watching my body change…”

**Social Isolation.** Falls also diminished participants’ ability to socialize with others, resulting in isolation, and feeling less an equal than a burden on friends and family. One participant (P16) described:

When I’m falling a lot, I tend not to go out as much… because it changes my relationships with people. When a person sees me fall … then … I have a relationship as an ‘injured.’ … I don’t want to feel like an invalid, or somebody that people have to take care of or look out for. That’s a tough feeling… when you’re with somebody who is at risk for a fall, then you’re no longer their comrade, they become your responsibility.

Even when they were willing to go out and socialize, many participants found they were limited as to where they could go with family and friends. One individual (P13) explained that when her extended family attended an annual festival that was held on dirt roads, the challenges of using a walker on uneven surfaces prevented her from joining in: “I tell you,… it’s a frustrating thing. Like when everybody goes somewhere, I can’t go because I can’t walk. When they go to [Festival Name]… it’s all rocks. I can’t roll [my walker] on those, you know?”

**Changing family roles**. Falls necessitated changes in familial roles and relationships: spouses and children became caregivers, and parents became dependent on children. One participant (P4) was totally reliant on her husband’s assistance: “Like when I need to take a bath at night… He’s here to do that for me [so I don’t fall]… he works only like four hours a day so he can be home with me to help me out.” Participants resisted this change in roles and relationships for as long as possible. One woman (P8) was very careful every time she moved and radically restricted her activities rather than asking others, particularly her children, for help: “I have my kids and all of that, but I don’t want to be… any more work or any trouble to anybody… that’s not necessary”.

Some family members adapted well to changing roles necessitated by falls. Others did not. Several participants described the willingness of family to pitch in and help with transportation, shopping needs, and to provide comfort, support and companionship. Some family members even changed their jobs or their work schedules to provide care. Other participants’ family members were unwilling to help, and some went so far as to become verbally abusive. One participant (P12) who was unable to do much of the housework reported that her son was angry with her because he thought she wasn’t pushing herself hard enough to do what she used to do. She cried as she explained, “My son… told me, ‘You don’t do nothing. Why don’t you go to a nursing home to die.’…”

**Diminished sense of self.** Falls profoundly eroded some participants’ sense of self. Prior to the onset chronic illness, participants felt that they had been independent, contributing members of their families and communities. Chronic illnesses and the falls associated with them had narrowed their worlds, forcing them to relinquish dreams and goals they once held. Reflecting on how altered family relationships diminished her sense of self, one woman (P12) stated: “That’s what kills me now… because I’m an independent person. I don’t like people to do things for me, and I don’t want to ask my kids to do things for me… that hurts a lot… I don’t like to lean on people… I like to clean my house, you know, but I cannot do all of these things. I can’t even take a broom to clean the floor….”

Many reflected on their inability to work as hard and accomplish as much as they once had. One man (P1) spoke about what his life had been like prior to the development of chronic illness and falls. Prior to his illness, “I was out all the time. The sun would come up and I was outside doing something; working on cars… working on restaurant equipment. Something. Now…[because of my falls]… I am unable to work… I used to live for my job. I loved working… Now, I’m sitting home … most of the day, just me and my dog.”

**Stigma.** Since the early days of the epidemic, those living with HIV have tried to manage disclosure of their status to avoid stigma. Many participants in this study did not want to fall around others because they felt that falling might mark them as having HIV. Falls were a marker of ill-health. If a person did not have an obvious reason for ill-health, such as cancer or older age, some participants believed that community gossip might lead to speculation about HIV. HIV has a history of physical signs that “out” an individual’s status to others. These include wasting and lipodystrophy. Some from this study would add falls to this list. They also did not talk with others outside of one or two family members or very close friends about their falls because of their fear that disclosure would lead to stigma.

Beyond affecting relationships at home, HIV-related stigma could also threaten a person’s job. One participant (P7) stated: “I just don’t want anybody to see (me fall) and think that I’m handicapped or that I have HIV… because I don’t want them to tell me I can’t work…”

Participants also feared stigma from providers—not from their HIV providers, but from those to whom they might be referred for specialty care. One participant admitted to not telling her primary care provider about her falls because she was afraid that her provider would send her to a specialist for an evaluation. She had already experienced stigma from one specialist and was afraid that this new specialist would also judge her negatively because of her HIV. As she stated (P11):

My doctor sent me to another doctor once because of my bone situation… he never turned around and talked to me. I said, “Doctor what’s the matter? Because every time I come here, you never even look at me.” I say, “you don’t care for me because I’m HIV positive?” He never even turned around. He … said “you came to me, I didn’t come to you.” I went back and told my doctor and she never sent me back.

But HIV was not the only source of stigma. Age and disability were other stigmatizing conditions. Although canes or walkers were frequently acknowledged as useful tools to help prevent falls, most participants were hesitant to use them as they marked them as being older or disabled. As one participant (P10) described: “I should be using a cane and I don’t use it… I don’t want to look like an old man.” Another (P7) reported, “that’s kind of a stigma, that you come to work with a cane… I don’t want anyone to think that I’m handicapped.”

## Discussion

In this qualitative study, we asked persons with HIV about the causes of their falls, what they did to prevent falls, how their providers responded to their falls, and how these falls impacted their lives. Many identified extrinsic causes for their falls, including slipping on icy sidewalks. Others identified intrinsic causes including HIV and its sequelae. Some focused on the interplay between intrinsic and extrinsic risk factors. Still others could not identify a discrete cause of their falls. Prevention efforts were undertaken only if the participant could identify a specific cause for their falls. For example, two participants felt that their falls were caused by their degenerating hip or knee joints, and believed that joint replacement surgery was the only way to definitively resolve their falls. Most providers were not concerned about falls once catastrophic causes were ruled out. Falls also held a variety of meanings for participants. There were routine falls that could happen to anyone. These were the falls caused by extrinsic risk factors. For some, falls with intrinsic causes were markers or reminders of suffering and loss, while for others they were a source of adaptation and strength.

Our research highlights that falls were an important concern for our participants because of their impact on participants’ lives. All of our participants had fallen multiple times. Some had never suffered a serious injury but were terrified of falling. Falls caused participants to restrict their activities, to lose their independence and self-reliance, and to experience profound changes in their relationships with friends and family. These outcomes have implications for the quality of life of our patients as they live longer with HIV.

Many of the causes of falls, approaches to prevention, and meanings of falls identified in our sample of relatively young HIV+ individuals reflect those identified in older populations [3]. Four important areas of similarity include fear of falling, viewing falls as a marker of aging and declining health, identifying falls as a source of stigma, and the importance of identifying a specific cause for falls in order to prevent them.

Among older adults in the general population, fear of falling is a common concern [29–34]. This fear is related to the physical consequences of falls such as fractures and death [34]. It is also related to fears of loss of independence, loss of physical capacity, loss of life, social embarrassment or stigma, and of being a burden to family members [29, 33]. Older adults often attempt to prevent falls by limiting their activities [32–34], being vigilant about where and how they walk, slowing down, and holding on to walls and railings [34]. Activity restriction has led to social withdrawal, social isolation, and altered relationships with family and friends [32]. Altered relationships have contributed to a loss of sense of self or a damaged self-identity [32]. These same concerns are reflected in the current study that includes a sample of much younger (mean age: 55 years) HIV+ individuals.

Many older adults also saw falls as a marker of aging [32, 35]. Participants in the current study also made this association. This is noteworthy as the average age of participants in this study was 55 years–an age not typically considered to be old. It was not always clear why participants associated their falls with aging, although some had seen older family members fall. Not only were falls a marker of aging but they were sometimes seen as a marker of death, or “the beginning of the end.” One participant associated his own falling with death because he worked in a nursing home and had seen his patients fall and die within a short period of time.

Falls are stigmatizing and a source of public embarrassment for older adults because they mark individuals as being old and frail [29, 32, 35]. This same association was evident in our findings. In addition, some participants also noted that they were afraid that their falls would lead people to find out that they were HIV+.

Research on older adults also suggests that identifying a specific cause for a fall helped to identify preventive actions. Falls attributed to chance or bad luck were not preventable [29, 32]. A similar attitude was espoused by some in the current study. If falls were associated with poor footwear, slipping on a wet floor, or even with an intrinsic cause such as alcohol use or polypharmacy, preventive actions could be taken. Falls that “just happened” were not preventable.

A number of participants in the current study reported adaptive responses to falls including hope, optimism and mastery. We did not find literature that spoke to this perspective among older adults.

The similarities between research on falls in older adults and in HIV+ individuals in the current study are important and concerning. Many of our participants, like older adults, viewed falls as a marker of declining health and illness. And yet the participants in our study were relatively young. If HIV+ individuals in their 40s and 50s limit their activities and social contacts because they are afraid of falling, then greater limitation, isolation, and disability will define how they age and will negatively impact their health and quality of life. An awareness of stigma related to aging could make them less likely to engage in preventive activities such as using a cane or walker.

There were also important differences in the responses to falls described by our participants versus those found in research conducted among older adults. Perspectives on normalizing falls and attitudes towards provider involvement were very different. In the literature, many older adults sought to normalize their falls, suggesting that frailty and falls were a normal part of aging. Among HIV+ participants, falls were not a concern if they were attributed to extrinsic factors (e.g. icy sidewalks or tripping on a rock while on a hike). However, falls attributed to intrinsic factors were abnormal and a marker of illness and decline; no one saw them as “normal”.

Older adults also resented provider “interference” in the guise of fall prevention efforts. They saw themselves as “fit, healthy, and able to manage,”[32] suggesting that they did not need help from providers to prevent falls. Many verbalized that while fall prevention interventions might be appropriate for “others,” they were neither appropriate nor necessary for themselves [30]. HIV+ individuals, in contrast, wanted their providers to be engaged and to share their concerns about falls and their sequelae and were frustrated by their inability to engage their providers in identifying fall prevention strategies.

These differences may suggest opportunities for fall prevention. HIV+ individuals may be more receptive to provider input regarding fall prevention. Therefore, engaging providers in fall prevention efforts is key. Understanding provider perspectives on falls in this population could create a foundation upon which interventions could be developed that would educate providers about falls, help them identify key fall risk factors, and understand concerns of patients. As demonstrated in the current analysis, listening to patient concerns and talking to patients in language that they understand constitute important components of fall prevention efforts.

The meaning that falls hold for HIV+ participants can be situated within the literature that has explored the meaning of chronic illness through the lens of illness narratives. Adaptation and suffering and loss are well-described responses to chronic illness in this literature [36–43]. Unfortunately, the literature does not describe how to help patients move from the perspective of suffering and loss to one of adaptation. This topic clearly requires further research, but useful directions for exploration include many possibilities. Fall clinics specifically for HIV+ individuals that address the multifactorial causes of falls and are sensitive to the unique features of falls in HIV+ patients (e.g. HIV-related stigma) is one potential approach. A multidisciplinary team would be an essential component of these clinics. A provider trained in geriatric care could provide a comprehensive fall assessment so that patients’ fall risk factors could be identified and a prevention plan put in place. A pharmacist could help with medication reconciliation and identification of medications that could be stopped to reduce polypharmacy. Nurses could provide patient education and follow-up. Social workers and other mental health specialists could help patients process their fall-related fears and identify strategies for addressing them. Substance abuse counselors would also be key team members. The development of fall support groups and/or buddy programs could provide peer support to those who fall and could be used to help participants identify management strategies and to decrease social isolation.

### Strengths and limitations

An important strength of our study is that our sample reflects individuals in the current HIV treatment era. The ART that is currently available is highly effective and easier to take than prior ART. As a result, patients are living and aging with HIV infection. While the number and classes antiretroviral medications have increased since these interviews (most notably, integrase inhibitors have become first-line medications), the fact that these medications are effective with minimal side effects has not changed. The concerns that our participants expressed are relevant to people currently living and aging with HIV, very different from those of the early days of the epidemic.

In addition, we allowed participants to choose how they would like to participate in the study: face-to-face with the interviewer in their home or office, face-to-face with the interviewer in an office on the medical school campus, or via telephone. By allowing patients to select the setting that was most comfortable for them, we enhanced the likelihood that we would capture rich narrative data on potentially sensitive subjects.

There were limitations to our study. We did not explore issues such as fall frequency or severity as we felt that these would be better addressed in a quantitative study. In addition, data for this study were collected at a single point in time, were based on participants’ retrospective description of their falls, and were thus vulnerable to recall bias. We attempted to minimize the effect of this bias by requiring participants to have had a fall no more than two years prior to the interview. Participants would not remember all slips or trips that occurred two years before the interview. However, they would likely remember significant falls, or falls with serious outcomes. We included individuals who had a variety of comorbidities in addition to HIV. While we explored the contribution of HIV infection on patient perspectives on their falls, we did not explore their perspectives on the role played by other comorbid illnesses. Participants in our study varied in age from 48 through 71 years. Only one participant was over the age of 65 years, the age group identified as being at risk for falls. This suggests that providers who care for HIV+ individuals may need to talk with their patients about fall-related concerns before these patients turn 65.

## Conclusions

Falls are an important concern for those HIV+ patients who fall. Future research must assess the knowledge, attitudes and practices of HIV providers regarding falls. Research is also necessary to identify causes of falls in HIV+ individuals and to develop predictive algorithms that can identify those most at risk for falls and related adverse outcomes such as injury, hospitalization, disability, and death. Interventions to address gaps in knowledge of providers are of key importance as is the development of interventions tailored to the specific needs and concerns of HIV+ individuals. These interventions, guided by a multidisciplinary approach, must take into consideration not only the causes of falls specific to this population, but also the meaning and impact that falls have in their lives.

## Supporting information

S1 Table COREQ checklist. (DOCX)
