# Correction: The beginning of the end: A qualitative study of falls among HIV+ individuals

**DOI:** 10.1371/journal.pone.0216192

**Published:** 2019-04-24

**Authors:** Julie A. Womack, Gina Novick, Terri Fried

The following information is missing from the Funding statement: This study was supported by Claude D. Pepper Older Americans Independence Center at Yale University School of Medicine (P30AG21342 National Institute on Aging).
